# Behavioural and pathomorphological impacts of flash photography on benthic fishes

**DOI:** 10.1038/s41598-018-37356-2

**Published:** 2019-01-24

**Authors:** Maarten De Brauwer, Luke M. Gordon, Tanika C. Shalders, Benjamin J. Saunders, Michael Archer, Euan S. Harvey, Shaun P. Collin, Julian C. Partridge, Jennifer L. McIlwain

**Affiliations:** 1 School of Molecular and Life Sciences, Curtin University, Perth, Australia; 2 Oceans Graduate School and the Oceans Institute, The University of Western Australia, Crawley, 6009 WA Australia; 3 School of Biological Sciences and the Oceans Institute, The University of Western Australia, Crawley, 6009 WA Australia

## Abstract

Millions of people take animal pictures during wildlife interactions, yet the impacts of photographer behaviour and photographic flashes on animals are poorly understood. We investigated the pathomorphological and behavioural impacts of photographer behaviour and photographic flashes on 14 benthic fish species that are important for scuba diving tourism and aquarium displays. We ran a field study to test effects of photography on fish behaviour, and two laboratory studies that tested effects of photographic flashes on seahorse behaviour, and ocular and retinal anatomy. Our study showed that effects of photographic flashes are negligible and do not have stronger impacts than those caused solely by human presence. Photographic flashes did not cause changes in gross ocular and retinal anatomy of seahorses and did not alter feeding success. Physical manipulation of animals by photographing scuba divers, however, elicited strong stress responses. This study provides important new information to help develop efficient management strategies that reduce environmental impacts of wildlife tourism.

## Introduction

Humans are fascinated by animals, and in addition to visiting zoos or aquaria and engaging in wildlife tourism, people spend a significant part of their time and money observing and photographing wildlife^1–3^. An estimated 700 million people visit zoos and aquaria annually, indirectly contributing more than US$350 million to conservation^2^. Wildlife tourism is estimated to be worth approximately £30 billion per annum (≈US$40 billion) and can provide a sustainable source of income to local communities, benefit conservation practices, and has the potential to educate the general public^1,4,5^. Tourism and photography are intrinsically linked, where photographs are used both to market destinations and record memories^6,7^. Where wildlife photography was traditionally the domain of large publishers like National Geographic, social media are now increasingly being used to share billions of wildlife and animal photos^3^. Despite the high number of human-animal interactions, the potential impacts of photography on animals remain unclear^8–10^. Animal welfare and ethics in tourism are frequently discussed in both the scientific and grey literature, yet very few studies have examined the behavioural or pathomorphological effects of photography on wildlife^1,11,12^.

Recreational scuba diving is an important sub-sector of wildlife tourism, with multiple studies highlighting its high economic value to local communities^13–15^. While scuba diving might indeed be less destructive than extractive activities, such as fishing, it does have potential environmental impacts. The impacts of scuba diving on fragile habitats such as coral reefs have received considerable research interest^16–18^. While the effects on habitat forming structures such as hard corals have been firmly established, far less is known about the effects on mobile fauna^19^. Studies on large-bodied species such as sharks have shown that diver interactions can increase an animals’ metabolism, cause behavioural changes, and reduce mobility^20–22^. However, little is known about the effects of scuba diving on smaller teleost fishes, although it has been established that diver presence can disturb spawning aggregations^8^ and that touching seahorses can lead to short-term behavioural changes^23^.

Overall, goal-oriented diver behaviour, such as photography, has greater impacts on the marine environment than general dive activities^24–26^. While taking pictures, divers spend more time close to marine life, causing damage to the substrate and often touching animals^27,28^. Divers will occasionally carry “muck sticks” to coax animals into a better position for taking photographs^18^. The effects of touching or moving marine life has not been studied in detail, but can be expected to cause behavioural changes^23,27^. The bright photographic strobes used in underwater photography frequently raise questions about potential impacts on animals’ behaviour and/or their visual systems, yet thus far, no significant effect of flash photography has been detected on the behaviour of teleost fishes^8,23^.

Despite the lack of scientific evidence, a multitude of regulations exist related to photographing marine wildlife based on the unsubstantiated concern of causing (temporary) blindness in animals, either while scuba diving or visiting aquaria. Public aquaria around the globe prohibit the use of flash while taking photographs, without any scientific evidence to support the ban. Scuba dive resorts in Southeast Asia often restrict the use of flash while photographing pygmy seahorses^29^ and in the U.K. a ban on using flash while taking pictures of seahorses is in place, despite open acknowledgment of a lack of evidence to support the ban^10^.

Charismatic and cryptic species such as seahorses (two families within the sub order *Syngnathoidei*) and frogfishes (family *Antennariidae*) are highly popular with underwater photographers and are often displayed in public aquaria^26,30^. Cryptic species such as these depend on camouflage to avoid predation. Many are slow swimmers not capable of fleeing from scuba diving photographers. Flash photography does not affect site persistence of seahorses, but touching them elicits, at the very least, short-term stress behaviours^23^. Species like seahorses are visual predators that rely on accurate resolving power to catch prey. Any reduction in visual acuity or sensitivity is likely to reduce survivorship^31^. The high intensity light of photographic strobe lights could theoretically result in phototoxic retinal damage. This damage could be either short term or permanent retinopathy due to photothermal, photomechanical and/or photochemical effects of high retinal irradiance. Retinopathy has been previously observed in mammals, including humans (e.g.^32,33^), and also in the photoreceptors of teleosts (e.g.^34,35^). However, a link between flash photography and damage to the eye structure of animals has yet to be shown. In addition, questions remain about the effects on fishes of scuba diver behaviour associated with flash photography, in particular the potential effects on feeding efficiency due to temporary reductions in visual acuity and other stress responses.

**Figure 1:**
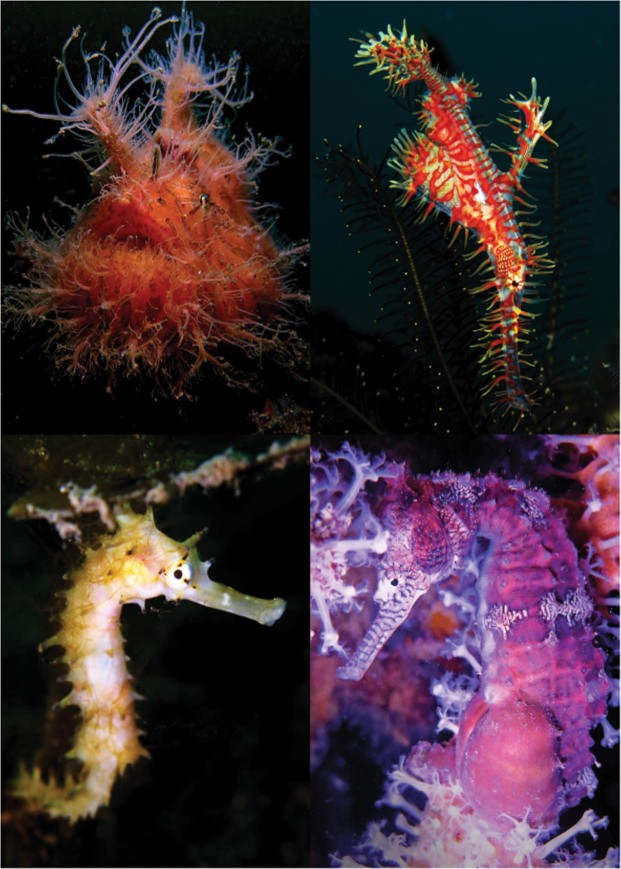
Four representative species used in this study (From top left, clockwise: *Antennarius striatus*, *Solenostomus paradoxus*, *Hippocampus subelongatus*, *Hippocampus histrix*).

To answer these questions, we conducted an *in situ* behavioural experiment on 13 species of teleosts from three families (*Syngnathidae*, *Solenostomidae* and *Antennariidae*) commonly found at dive sites throughout Southeast Asia (Fig. 1). We then ran two controlled aquaria experiments to assess the behavioural and pathomorphological effects of flash photography on a species of seahorse. Specifically we set out to: (1) Quantify the effects of diver behaviour associated with flash photography on slow-moving, cryptic fishes; (2) Assess the effects of photographic flashes on the Western Australian seahorse (*Hippocampus subelongatus*) and (3) Examine the pathomorphological impacts of photographic flashes on the ocular and retinal anatomy of *H*. *subelongatus*.

## Results

### Diver effects on fish behaviour

During this experiment, 82 different individual fish were observed (Table 1). Fish that responded to the photographing diver by moving greater than 50 cm away were not observed frequently enough to include in the analysis. Therefore, all further references to fish movement refers to animals moving less than 50 cm away from the diver.

**Table 1:** Species (N) observed during the diver behaviour effects field experiment.

Family (N)	Genus (N)	Species (N)	TP (N)	T1 (N)	T2 (N)	T3 (N)
*Antennariidae* (48)	*Antennarius* (*36*)		**9**	**9**	**9**	**9**
		*commersoni* (*2*)	1	1	0	0
		*pictus* (*27*)	7	5	8	7
		*striatus* (*6*)	1	2	2	2
		*sp*. (*1*)	0	1	0	0
	*Antennatus* (*12*)		**3**	**3**	**3**	**3**
		*sp*. (*ocellated*) (*10*)	1	3	3	3
		*nummifer* (*1*)	1	0	0	0
		*sp*. (*1*)	1	0	0	0
Syngnathoidei* (34)	*Hippocampus* (*20*)		**5**	**5**	**5**	**5**
		*histrix* (*8*)	1	3	2	2
		*barbouri* (*5*)	3	1	1	0
		*kuda* (*6*)	1	0	2	3
		*alatus* (*1*)	0	1	0	0
	*Solenostomus* (*14*)		**3**	**4**	**4**	**3**
		*paradoxus* (*4*)	0	0	2	2
		*cyanopterus* (*10*)	3	4	2	1

*Syngnathoidei is a suborder, containing sister-families *Syngnathidae* and *Solenostomidae*. TP: Diver presence treatment; T1: Flash only treatment; T2: Manipulation without flash treatment; T3: Manipulation including flash treatment. Every individual animal was also observed from >2 m distance as a control (N = 82).

#### Antennariidae

**Figure 2:**
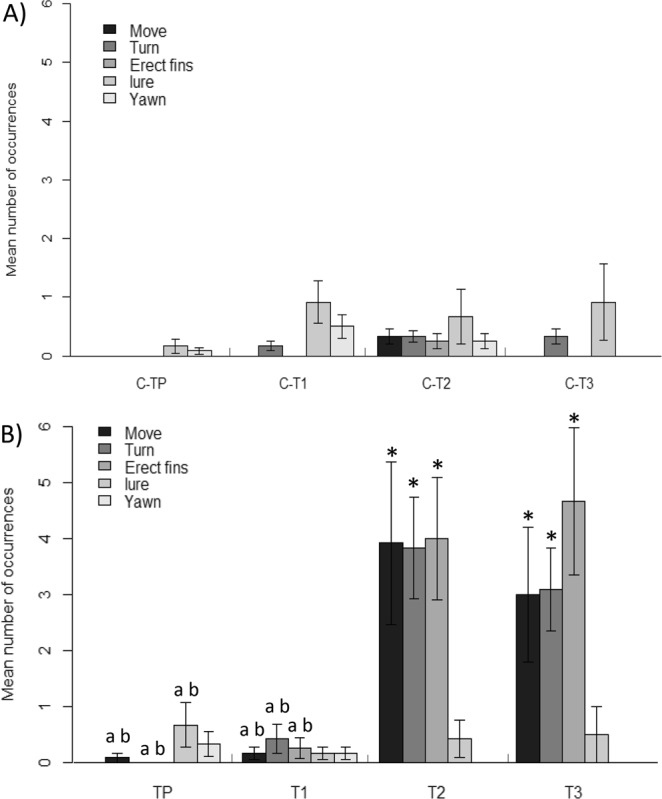
Mean number of occurrences of different reactions (±SE) of *Antennariidae* from the central Philippines to diver presence and flash photography. (**A**) Control observations. (**B**) Treatment observations. TP: diver presence (N = 12), T1: flash (N = 12), T2: manipulation (N = 12), T3: manipulation + flash (N = 12). Significance level of treatments compared to control after Holm-Bonferroni corrections: p < 0.05, *different to Control, ^a^different to T2, ^b^different to T3.

Insufficient instances of feeding were observed in individuals of this family of teleosts, so this variable was not included in further analysis. Since no statistically significant effect of genus was found, data were analysed at only the family level. Diver presence (TP) and flash (T1) treatments had no significant effect on any of the behavioural reactions (Fig. 2, Table in Supplementary Materials). Manipulation (T2) and manipulation + flash (T3) significantly increased the occurrences of movement, turning away from the stressor and erecting fins (Fig. 2, Table in Supplementary Materials). None of the treatments had a significant effect on luring or yawning (Fig. 2, Table in Supplementary Materials). The four groups of paired control observations per treatment were not significantly different from each other.

There were significant differences between the treatments for movement, turning, and erecting fins (Table 2). T2 and T3 both had a greater number of occurrences of all of these, and were different from TP and T1, but not from each other (Table 2). TP and T1 were never different from each other (Table 2).

**Table 2:** Results Kruskal-Wallis rank sum tests comparing different treatments (excluding controls) and for post-hoc pairwise Wilcoxon rank sum tests.

Reaction	Kruskal-Wallis			Wilcoxon	
	Χ^2^	*df*	p	TP (p)	T1 (p)
*Antennariidae*					
*Movement*	15.427	3	0.002**	T2 (0.003**)	T2 (0.006**)
				T3 (0.008**	T3 (0.018)
*Turn*	25.771	3	<0.001***	T2 (<0.001***)	T2 (<0.001***)
				T3 (<0.001***)	T3 (0.003**)
*Erect*	23.403	3	<0.001***	T2 (<0.001***)	T2 (0.002**)
				T3 (<0.001***)	T3 (0.002**)
*Yawn*	4.280	3	0.233	—	—
*Lure*	1.047	3	0.790	—	—
*Syngnathoidei*					
*Turn*	6.364	3	0.095	—	—
				—	
*Feed*	4.791	3	0.188	—	—
*Hippocampus* spp.					
*Movement*	7.287	3	0.063	—	—
				—	—
*Solenostomus* spp.					
*Movement*	4.239	3	0.237	—	—

For Wilcoxon tests (excluding controls), only significantly different treatments are shown. TP and T1 were never different from each other, neither were T2 and T3. TP: diver presence, T1: flash, T2: manipulation, T3: manipulation + flash. Significance level after Holm-Bonferroni corrections: *p < 0.05, **p < 0.01, ***p < 0.001.

#### Syngnathoidei

**Figure 3:**
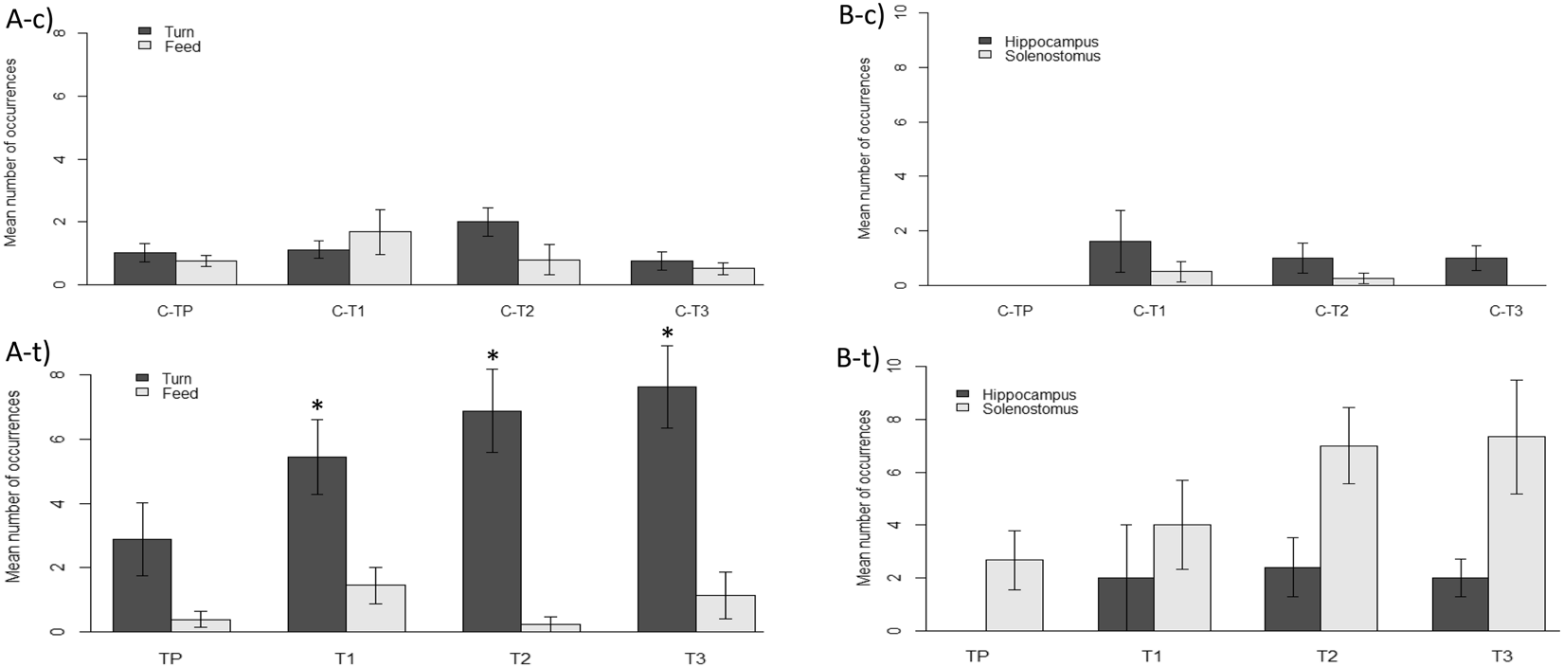
(**A**-**t**) Mean number of turning and feeding reactions (±SE) of *Syngnathoidei* to different treatments; (**A**-**c**) Paired control observations. TP: diver presence (N = 8), T1: flash (N = 9), T2: manipulation (N = 9), T3: manipulation + flash (N = 8). (**B**-**t**) Mean number of movement reactions (±SE) of *Hippocampus* spp. and *Solenostomus* spp. to different treatments. (**B**-**c**) Paired control observations. *Hippocampus*: TP (N = 5), T1 (N = 5), T2 (N = 5), T3 (N = 5). *Solenostomus*: C (N = 14), TP (N = 3), T1 (N = 4), T2 (N = 4), T3 (N = 3). Significance level of treatments compared to control after Holm-Bonferroni corrections: p < 0.05, *different to Control.

When testing differences between taxa, *Solenostomus* spp. and *Hippocampus* spp. did not react significantly different for turning away (p = 0.738) or feeding (p = 0.075), but there was a genus-effect for increased movement (p < 0.001). Data for movement were therefore analysed at the genus level and data for turning and feeding were analysed on the family level. On the family level, there was no effect of treatments on feeding (Fig. 3, Table in Supplementary Materials). *Syngnathoidei* did show a significant increase in turning for all treatments, except for diver presence (Fig. 3, Table in Supplementary Materials). Kruskal-Wallis tests showed that for *Syngnathoidei*, *Hippocampus* spp., and *Solenostomus* spp. there were no differences in the reactions to any of the different treatments (Table 2). The four groups of paired control observations per treatment were not significantly different from each other.

### Behavioural effects of photographic flashes

A total of 47 control, 48 low frequency, and 46 high frequency trials were analysed. One seahorse in the high treatment group was affected by pouch emphysema after the second repetition and was subsequently removed from analyses as its capacity to swim had become compromised. One video failed to record during the first repetition of the control treatment and could not be analysed. Repeated measures ANOVAs did not show a statistically significant difference between repetitions, sex, or size for any of the variables. Therefore, data of the four different repetitions were combined and analysed together.

#### Hunting efficiency

Flash treatments had no significant effect on the time seahorses spent hunting (p = 0.796). The number of strikes at prey was not different between treatments (p = 0.965), neither was the catch success rate (p = 0.147).

#### Spatial use

χ^2^ tests established that there was no significant effect of treatment compared to the control on the time seahorses spent in different zones of the tanks (χ^2^_(6)_ = 6.470, p = 0.373). The way seahorses oriented themselves in the tanks did not differ significantly between control and treatments (χ^2^_(4)_ = 2.180, p = 0.701).

#### Activity

**Figure 4:**
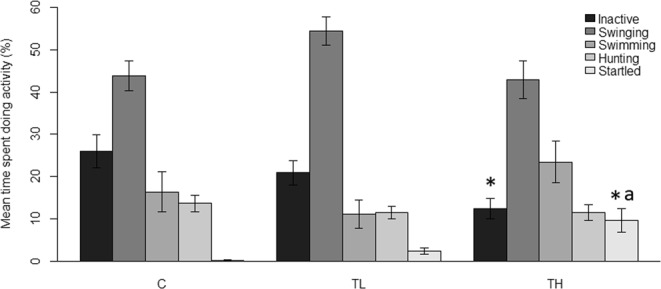
Mean time (±SE) seahorses spent doing different activities during different flash treatments. C = Control (N = 47), TL = Low frequency (N = 48), TH = High frequency (N = 46). Significance level of treatments: p < 0.05, *different to C, ^a^different to TL.

Repeated measures ANOVA confirmed a significant effect of treatment on the time seahorses spent inactive (p = 0.028) and showing startled responses (p < 0.001). There were no differences in the times spent swinging, hunting, or swimming. The control group spent more time being inactive compared to high treatment (p = 0.018) seahorses, but not compared to the low treatment group (p = 0.770). Inactivity was not different between low and high treatment groups (p = 0.106). Startled responses in control and low treatment groups were not different from each other (p = 0.119), but both were different from the high treatment group (C – TH: p < 0.001; L – TH: p = 0.024) (Fig. 4).

#### Ventilation rate

**Figure 5:**
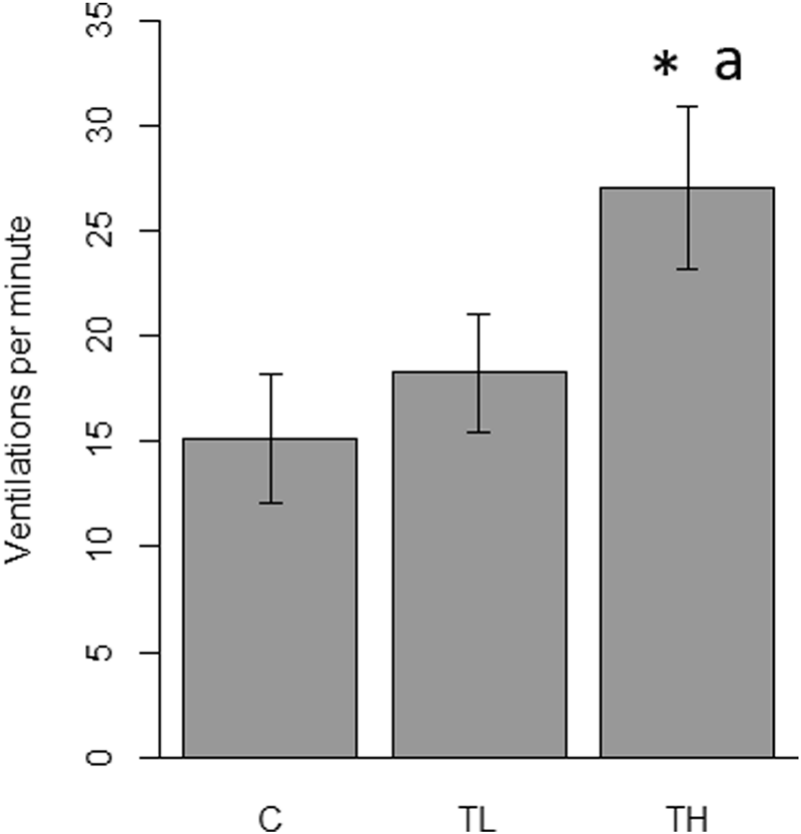
Seahorse ventilation rates (±SE) during different flash treatments. C = Control (N = 47), TL = Low frequency (N = 48), TH = High frequency (N = 46). Significance level of treatments: p < 0.05, *different to C, ^a^different to TL.

Treatment had a significant effect on the ventilation rates of the seahorses (p < 0.001). Ventilation rate of high treatment seahorses (27 beats min^−1^) were significantly higher than control (15 beats min^−1^) and low (17 beats min^−1^) treatment groups (C – TH: p < 0.001; L – TH: p = 0.006). There was no difference between control and low treatment groups (p = 0.439) (Fig. 5).

### Pathomorphological effects of photographic flashes

Multivariate PERMANOVA analysis showed no significant effect from treatment on gross ocular morphology (Pseudo-F_(1,18)_ = 0.58, P = 0.65) or retinal morphology (Pseudo-F_(1,18)_ = 0.50, P = 0.79). Individual t-tests showed no significant differences between any variables in the control group and the flash treatment group (Table in Supplementary Materials). No lesions, oedema or visible changes were observed in the retina at the light microscopy scale. We found no evidence that photographic strobes result in changes to gross eye anatomy (shape or size of the eye and/or lens) or basic retinal morphology defined here as the thickness of the whole retina or the thickness of each retinal cell layer.

## Discussion

Wildlife tourism is important for supporting livelihoods worldwide, but potential impacts caused by photography or photographer behaviour need to be minimised to ensure sustainability and for best practice in animal welfare. This study showed that repeated photographic flashes delivered over a period of 34 days and involving 4600 exposures to full power flash gun discharges per animal did not cause overt changes in ocular morphology or retinal gross anatomy, such as the thickness of retinal layers or photoreceptor size, in *Hippocampus subelongatus*. More importantly, these flashes had no observable impact on foraging behaviour or feeding success rates in a species that is known to rely on vision to capture small moving prey and has relatively high spatial resolving power^31^. Manipulation of animals by photographing divers in the wild, however, elicited very strong flight and stress responses. These results provide important new information for the development of best-practice photography guidelines for wildlife tourism.

Tourism management bodies including those in government organisations and public aquarium facilities, as well as tourism operators, have developed rules and regulations restricting the use of flash guns or strobes, while photographing a range of animals^10,29^. These well-intended preventive measures have not been based on scientific evidence. This experiment demonstrates that repeated photographic flashes do not appear to cause gross retinal damage in the seahorse *Hippocampus subelongatus*, at least over the duration of this experiment and under these conditions. In this study, we used a strobe at a higher intensity (double to triple) than is usually applied when photographing seahorses *in situ* underwater, and at a much higher intensity than could be reached by compact or phone cameras in an aquarium. In addition, phototoxicity is more frequently associated with extended exposure to intense light sources, in contrast to the very short exposures typical of photographic flashes^32,33^ which suggests that flash exposures may be less likely to cause retinal damage.

Caution remains necessary, as different species may well have different susceptibility to photic damage. The species of seahorse studied here, *Hippocampus subelongatus* inhabits relatively low-light environments^31^ so it may be relatively more susceptible to photic injuries when compared to more shallow living species and could therefore be considered a conservative model species for these tests. Fish inhabiting surface waters, for instance, where they are subject to high intensity caustic images of the sun focused by surface waves and wavelets^36^ may well be more resistant. This near-surface ‘flicker’ results in short duration (ms) increases in irradiance of 10 fold or more^37,38^. When underwater flicker is considered in terms of a 3-dimensional radiance distribution, fluctuations in intensity can be even higher, up to 100 times^38^, and, when focussed on the retina, highly localised, high intensity illumination will result. Animals with wide fields of view and those inhabiting sunlit shallow waters will therefore be likely to have mechanisms to counter photic retinal damage, whereas other species in less variable and less bright habitats than *H*. *subelongatus* may be more susceptible.

Our field experiment demonstrated that cryptic fishes are most strongly affected by diver manipulation. The highly significant increase in movement for frogfishes, species which rarely move if undisturbed, implies a considerable energy expenditure which could lead to decreased fitness^39^. Movement reactions differed between *Solenostomus* spp. and *Hippocampus* spp., reflecting different defence mechanisms used by each family. Seahorses are less mobile than ghostpipefishes (*Solenostomus* spp.) and rely more on camouflage than on flight response. When divers manipulated animals there was no difference whether or not the diver also used flash. In most cases, flash photography had no more effect than diver presence.

We demonstrated that the argument that flash photography might negatively affect feeding behaviour due to temporary blindness caused by flash photography does not hold up for the species tested in this study. Neither the field nor tank experiment yielded a decrease in the time spent hunting or in feeding efficiency in *H*. *subelongatus*. Even in the treatments that caused movement reactions, feeding rates were unchanged, indicating that despite potential distress, visual acuity was not impacted. Similar results have been observed when testing the effects of temperature stress on *H*. *guttulatus* where food intake in seahorses was not decreased despite increased ventilation rates^40^.

High flash treatment caused similar increases in ventilation rates, indicating seahorses might have experienced stress. It remains unclear if this increased ventilation was caused by the observed increase in movement, or how strong this stress reaction was and if the animals were indeed stressed. While ventilation rate can be used as a proxy for stress, it does not always reflect the strength of the stimulus^41^. However, increased gill ventilation rates in animals experiencing high flash exposure, regardless if caused by stress or through increased movement, suggests increased metabolic rates, which, if sustained, would have consequences for food requirements. In the case of photographic flash, the direct effects seem to be relatively small and were likely exacerbated by seahorses being kept in captivity without the possibility of escaping the stressor. While scuba diving, the reactions seen in the tank study would likely translate to the animal fleeing.

While the tank study indicates that seahorses might experience some discomfort caused by photographic flash, the behavioural effects seen during the tank experiment are negligible compared to effects caused by diver presence and manipulation in a scuba diving setting. This is consistent with another study on a similar-sized seahorse species (*H*. *whitei*), which showed similar short term movement in response to handling and no differences between flash photography and diver presence^23^.

It remains unknown if repeated exposure to photography over periods of months or years could lead to chronic stress and associated pathology in cryptobenthic fauna^39,42^. Previous studies have found that minimal exposure to photographing divers did not change seahorse site persistence^23^, and did not increase stress levels in Ram cichlids or Mozambique tilapia^43,44^. It does, however, remain unknown what the behavioural or physiological effects would be from being manipulated by up to 50 divers per day, as is the case in popular dive sites (personal observation MDB). Further work, including specific studies of retinal cell apoptosis (e.g.^45^), transmission electron microscopy studies of photoreceptor and retinal pigment epithelial cell ultrastructure, electrophysiological studies of photoreceptor function, and molecular approaches, would be required to completely eliminate the possibility of light-induced retinal damage in seahorses exposed to underwater strobe lights, but these were beyond the scope of this study.

This study has important implications for dive tourism and public aquaria. It may not be necessary for public aquaria to enforce a ban on flash photography, provided tanks are large enough for animals to move away from the stressor. Popular exhibits might still want to avoid flash photography to prevent animals retreating out of view. For scuba diving, the results of this study clearly show that divers should avoid touching or pursuing animals, rather than focusing on regulations on flash use that have no scientific basis.

## Conclusion

This is the first study to investigate the combined pathomorphological and behavioural impacts of photographer behaviour and photographic flashes on animals. We conclude that the effects of photographic flash alone are minor and do not have a stronger impact than those caused by human presence or photography without flash. However, manipulating animals during photography elicits very strong evasive responses and should therefore be avoided. While feeding efficiency was not negatively impacted in this study, repeated diver manipulation in highly popular dive sites could still have the potential to lead to chronic stress, increased energy requirements, and reduced fitness in photographed animals.

## Materials and Methods

### Ethics statement

All experiments were conducted in in compliance with the Australian Code for the Care and Use of Animals for Scientific Purposes and were approved by the Animal Ethics Committee of Curtin University (AEC_2016_29) and The University of Western Australia (RA/3/100/1220). Seahorse collecting in Western Australia was conducted under Fisheries Exemption Number 2798, approved by the Western Australian Department of Fisheries.

### Effects of diver behaviour

#### Site description

To investigate the effects of disruptive diver behaviour *in situ*, a field experiment was conducted in Dauin, Philippines (9° 11′ 19″ N, 123° 16′ 10″ E). Dauin is an increasingly popular SCUBA diving destination for observing and photographing cryptobenthic fauna^15^. Experiments were conducted across five sites spanning 2 km. Bottom composition on all sites was predominantly soft sediment (volcanic sand), with very limited seagrass or coral growth in shallow areas.

#### Species description

For this study, we observed species of *Antennariidae* and *Syngnathoidei*. *Antennariidae* (frogfishes) are ambush predators that use an adapted first dorsal spine to attract prey and occur on shallow coral reefs and soft sediment sites (Pietsch and Grobecker 1987). The sub-order *Syngnathoidei* contains the *Syngnathidae* and *Solenostomidae* families, which are considered to be sister families^46^. Both families are visual ambush predators that feed on small invertebrates, ingested through a tubular mouth. The *Syngnathoidei* species used in this study were medium-sized (Range: 50 mm–150 mm Total Length (TL)) and were found near seagrass, plant debris or rocks.

#### Experimental design

Experiments were conducted by scuba diving in May 2016, opportunistically sampling across five sites. Care was taken to target different depths and areas between different dives to avoid re-sampling the same individuals within the same site, an approach that is possible due to the limited mobility of the target species. Observations were only taken in the daytime (between 9:00 am and 4:00 pm) and no deeper than 27 m. When a focal animal was found, each individual was initially observed for three minutes from a minimum 2 m distance (Control - C). Preliminary observations showed this distance did not affect individual behaviour, which is in line with previous research that found that small fish are less shy than larger fish^47^. After the control observations were recorded, the animals were randomly allocated to one of four experimental treatments conducted by a second researcher and adapted from Harasti *et al*.^23^: diver presence (TP), diver with flash (T1), diver manipulation without flash (T2), or diver with flash + manipulation (T3). The diver presence treatment (TP) consisted of a diver closely approaching the animal (<30 cm), while holding a DSLR camera and remaining at this distance for three minutes without taking pictures or touching the animal. This treatment is considered to be similar to no-flash photography or observing without a camera^23^. Flash treatment (T1) consisted of a diver with a DSLR camera approaching the animal closely (<30 cm), remaining near the animal for three minutes, and taking a total of 15 pictures using both flash strobes. During the manipulation treatment (T2) the diver carrying the DSLR camera approached the animal closely (<30 cm) and remained at that distance for three minutes. Instead of taking pictures, the researcher gently nudged the animal 20 times using a 30 cm “muck stick” (a handheld stainless steel or aluminium rod). This type of manipulation is common amongst underwater photographers and is used to reposition an animal in order to get a better picture^18^. The flash and manipulation treatment (T3) combines T1 and T2, with the researcher staying close to the animal (<30 cm) for three minutes, taking 15 pictures with flash and gently prodding the animal 20 times. This treatment is the equivalent of the behaviour of photographers who take pictures, while manipulating the animal. For each treatment, the researcher who undertook the initial control observation (C) recorded the responses, while staying a minimum distance of 2 m away from the focal animal and the second researcher conducting the experimental treatments. The camera used during the experiment was a Canon 7D Mk1, with a Nauticam housing and two external Inon Z240s high power (ISO 100 Guide Number 24) strobes. Strobes were fired at half strength, with a light colour temperature of 5500 K.

#### Response categories

Changes in the behaviour of focal animals were recorded continuously and classified as one of three categories: avoidance behaviour, threat displays, and feeding behaviour. Avoidance behaviour was further specified as: turning to face away from observer, moving less than 50 cm, and moving more than 50 cm. Threat displays were defined as: erecting fins (frogfishes only) and yawning (frogfishes only). *Syngnathoidei* did not show threat displays. Feeding behaviour was further categorised as: waving lure (frogfishes only) and feeding (striking at prey).

#### Data analyses

Differences in behavioural responses were analysed separately per family as certain responses are family-specific (e.g. Syngnathidae are physically incapable of yawning). To ensure behaviour was similar for different taxa, preliminary Kruskal-Wallis tests were conducted comparing the different taxa, taxa which differed significantly from each other in their reactions were analysed separately.

The analysis tested two separate questions: (1) if different diver behaviour caused a behavioural change compared to having no diver present; and (2) if, in the presence of a diver, different treatment caused different reactions. To answer the first question, the control observations (conducted for each individual) were compared to treatments using paired Wilcoxon rank sum tests, as data was non-normally distributed. Kruskal-Wallis rank sum tests were then carried out to answer the second question, i.e. to detect differences in reactions between treatments. The individual control observations were excluded from this analysis, in order to only test the different diver treatments. The four groups of paired control observations were compared using Kruskal-Wallis rank sum tests to check for differences in control groups. Statistically significant effects were investigated using pairwise Wilcoxon rank sum tests. Holm-method p-value corrections were applied and used when testing for significance to reduce the chances of type I errors^48^. Original non-corrected p-values are presented in results for clarity, as non-significant Holm-adjusted p-values commonly resulted in p-value equal to one. All data analyses were conducted using the R software package^49^.

### Behavioural effects of photographic flashes

To test the effects of photographic flashes independent of diver presence, an aquaria experiment was conducted at the Curtin Aquatic Research Labs in Perth, Australia. The experiment ran for 6 weeks from September to October 2016. The West Australian seahorse (*Hippocampus subelongatus*), a medium-sized seahorse species endemic to Western Australia which is relatively abundant in the waters near Perth^50^, was used as a model species. This species was used rather than the species observed in the field experiment, as the latter could only be acquired via the marine aquarium trade without guarantees of sustainability or non-destructive catch methods.

#### Specimen collection and husbandry procedures

Three 192L tanks (122 cm × 35 cm × 45 cm) were set up four weeks prior to collecting seahorses to establish stable water quality conditions. Each tank had its own recirculating system and artificial seagrass made of rope placed to serve as holdfasts for seahorses. The tank room had a regime of 14 hours artificial light per day, mimicking local daylight hours at the time. Water temperature, O_2_ saturation, salinity, ammonia, and nitrites were tested daily to ensure optimal conditions. Water temperature was kept between 17 °C and 19 °C and salinity maintained between 36 ppm and 38 ppm. Tanks were cleaned twice per week and 25% water changes performed once every three days, or as required. Seahorses were fed three times daily with live *Artemia* enriched with a commercially prepared emulsion of essential fatty acids. Fish were left to acclimatise in the holding tanks for three weeks prior to the start of experiments.

Once conditions in the holding tanks were stable, 37 seahorses of a size range between 110 and 240 mm TL were collected by scuba diving from four different sites along the Perth shoreline. Care was taken not to collect pregnant males or mating pairs. Prior to placing seahorses in holding tanks, each seahorse was tagged using small, subcutaneous elastomer tags to identify individuals^51^ and TL measured as the sum of head, body and tail length^52^. Seahorses were then placed in one of the three tanks independent from treatment, to avoid any potential holding tank effects.

#### Experimental design

**Figure 6:**
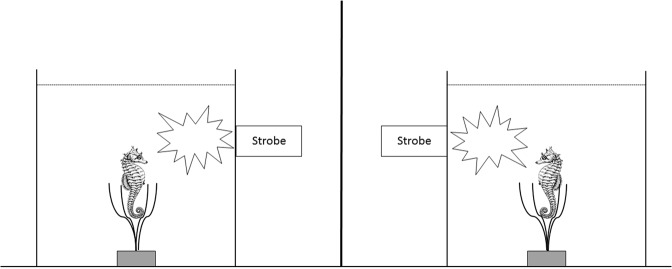
Treatment tank setup: two 17L tanks separated by a black cloth, a holdfast consisting of artificial seagrass in the middle of each tank, a Sea&Sea YS-250PRO underwater strobe placed against each tank. A video camera was placed in front of both tanks and a black cloth was hung around the tanks.

Each seahorse was allocated to one of three treatment groups: control (C) (N = 12), low frequency (TL) (N = 12) and high frequency (TH) (N = 12). During the experiment, seahorses were removed from their holding tanks by hand and placed individually in one of two 17L treatment tanks (31 cm × 23 cm × 25 cm), with an artificial holdfast similar to those in the holding tanks placed in the centre. A Sea & Sea YS-250PRO underwater strobe (Colour temperature 5600 K; ISO 100 full power guide number 32) was placed on the side of each treatment tank, touching the glass of the tanks (Fig. 6, Supplementary Materials). A black cloth was hung around each of the treatment tanks to avoid any observer effect and to avoid seahorses seeing each other. Water in the treatment tanks was the same temperature and salinity as water in the holding tanks. A small desk lamp was permanently placed 1.5 m above the tanks to provide sufficient light for filming trials. Seahorses were left to acclimatise in the treatment tanks for five minutes prior to commencing treatment. A Sony Handycam HDR-CX12 video camera was placed in front of each treatment tank to record seahorse behaviour. Trials ran in the morning, prior to feeding the seahorses.

Each trial started by releasing 2 ml of seawater containing *Artemia* (approx. 25 *Artemia*) into the treatment tank using a pipette, after which trials ran for ten minutes. In control treatments, seahorses were left undisturbed. For low frequency treatments, the strobes were fired at highest strength once every 30 seconds. In the high frequency treatments, strobes were fired at highest strength once every 15 seconds. By using the highest flash strength and placing the strobes against the tank, flash intensity is stronger than in normal scuba dive photography, where animals the size of seahorses are usually photographed with strobes on mid-or one third of maximum strength. The strobes we used were also of much higher intensity than the built-in strobes of cameras frequently by visitors in zoos or aquaria. Strobes were fired remotely by a researcher on the other side of the black cloth, out of view of the seahorses (example video of trial available in Supplementary Materials).

After the trials, seahorses were put back into their holding tanks, the water in the treatment tanks was changed and two new seahorses were placed in the treatment tanks. The experiment ran for three consecutive days until each seahorse had received its treatment. Trials were repeated on each subject a total of four times, during which each seahorse remained in their designated treatment group. Between trials, seahorses had a minimum of four days recovery time without exposure to any treatment to minimise stress and avoid any habituation effects. Upon conclusion of the experiment, seahorses of the low frequency treatment group and two seahorses of both the high and control groups were donated to a local public aquarium, as releasing animals back in the ocean was not possible due to permit restrictions.

#### Response categories

Behavioural response categories were adapted from^53^. Four response categories were used: Hunting efficiency, spatial use, activity, and ventilation rate (for definitions see Table 3). Hunting efficiency counted the instances of a seahorse striking at food, catching food, and hunting success (ratio of catches to strikes). Spatial use was measured in two ways: the distance of the animal from the side of the tank where the strobe was positioned, and the seahorses’ orientation towards the strobe. Activity was the time seahorses spent showing a specific behaviour: inactive, swinging, swimming, hunting, or startled responses. Ventilation rates were used as a proxy for stress and were measured by counting and averaging opercular beats twice for two periods of 20 seconds.

**Table 3:** Definitions of seahorse responses to flash treatments.

Category	Action	Description
Hunting efficiency		
Action	*Strike*	Seahorse attempts to strike at prey.
	*Catch*	Seahorse catches and swallows prey.
	*Success*	Ratio of catches to strikes.
Spatial Use		
Position	*Zone 1*	The first vertical quarter of the tank, closest to the strobe.
	*Zone 2*	The second vertical quarter of the tank relative to the strobe (Holdfast present in this zone).
	*Zone 3*	The third vertical quarter of the tank relative to the strobe (Holdfast present in this zone).
	*Zone 4*	The forth vertical quarter of the tank furthest away from the strobe.
Orientation	*Towards*	The seahorse faces in the direction of the strobe.
	*Neutral*	The seahorse faces towards the camera or away from camera, both eyes are visible (30 degree angle towards or away from camera).
	*Away*	The seahorse faces away from the strobe.
Activity		
Rest	*Inactive*	The seahorse remains resting, without performing any kind of movement, while attached or unattached to the holdfast.
	*Swinging*	The seahorse remains attached to the holdfast, with slight movements of the head or body.
Active	*Hunting*	The seahorse swims or tilts the body, head or snout in the direction of prey. Attempted strikes at food irrespective of success.
	*Swimming*	The seahorse moves horizontally or vertically (through the water or across the bottom of the tank) with undulating pectoral and dorsal fins.
	*Startled*	The seahorse repeatedly and rapidly contracts and contorts the body, strikes or swims into the tank wall, displays sudden erratic movements.

#### Data analyses

Videos of the trials were analysed twice (see Supplementary Materials for example of video). During the first analysis, hunting efficiency and spatial use (noted every 10 seconds) were measured. To measure position accurately, treatments tanks were divided in four vertical, equal-sized zones marked by strips on the outside of the aquaria. The second analysis was used to measure seahorse activity (noted every 10 seconds) and ventilation rates (measured at 5 minutes and 10 minutes, then averaged). Video analysis started from the moment *Artemia* were introduced into the treatment tanks and lasted for ten minutes. Videos were analysed in a random order and behavioural definitions reviewed frequently to minimise observer drift effects^54,55^.

Data on behavioural responses were transformed to best meet assumptions of normality before statistical analyses that were conducted in R^49^. We used a repeated measures ANOVA to detect the effects of treatments on behavioural responses, with seahorse ID as a random factor to examine the effect of different replications. ANOVA’s were followed by Tukey’s HSD post hoc tests for significant effects. We further tested for interactions between seahorse sex, size, and origin. Spatial use was tested using *χ*^2^ contingency table tests. All analyses were conducted in R using the lme4 package^56^ for the repeated measures ANOVA and the multcomp package^57^ for performing the post-hoc tests.

### Pathomorphological effects of photographic flashes

To assess the pathomorphological effects of intense flash photography on the visual system of cryptobenthic fauna, seahorses from experiment two were subjected to a second treatment. Ten randomly selected seahorses from both the high frequency treatment (TH) and control (C) groups were retained after concluding experiment 2. Seahorses were held in the same holding tanks as experiment 2 for the duration of this experiment.

#### Experimental design

Seahorses were moved daily into one of two treatment tanks (61 cm × 25 cm × 31 cm). All seahorses (N = 10) of the control group were placed in a control tank, and all seahorses (N = 10) of the high frequency treatment were placed in a flash treatment tank. Two Sea&Sea YS-250PRO underwater strobe (Colour temperature 5600 K; ISO 100 full power guide number 32) were positioned on either side of the flash treatment tank, touching the glass of the tank. No strobes were placed next to the control tank. Both tanks were separated from each other by black cloth to avoid flash reaching seahorses of the control group. Seahorses were kept in the treatment tanks for 150 minutes, during which the strobes of the flash treatment tank were fired a total of 200 times at highest strength. While this strength would not normally be used during underwater photography, this was designed to test the “worst-case scenario”. Strobes were fired remotely by a researcher on the other side of the black cloth, invisible to the seahorses. Over the course of both flash experiments (34 days), seahorses in the highest flash treatment were subjected to a total of 4600 flashes or an average of 135 day^−1^. The experiment ran for 15 days, after which seahorses were euthanized with a lethal overdose of tricaine methanesulfonate (MS 222).

#### Eye and retina preparation

After euthanasia in MS222, seahorse eyes (both left and right) were enucleated and both the anteroposterior and dorsoventral diameters of the eye were measured with a pair of digital callipers to the nearest 0.01 mm. The anterior segment (cornea, lens and iris) were then dissected free of the scleral eyecup and the lens diameter also measured using the callipers. All ocular and retinal measurements (see below) were performed “blind”, where the experimenter was not aware of which individual belonged to each treatment group.

Each eye was then immersion fixed in 2.5% glutaraldehyde, 2% paraformaldehyde in 0.1 M phosphate buffer (pH 7.2) and stored in fix at 4 °C for four weeks. The material was then washed in 0.1 M phosphate buffer and post-fixed in 1 to 2% osmium tetroxide. The eyecup was then embedded in Araldite, and semi-thin (1 µm) sections were cut on an ultramicrotome (LKB) using glass knives. Semi-thin sections were stained with Toluidine blue, coverslipped in Entellan, mounting medium and photographed as TIFF images using an Olympus DP30 low noise 12-bit monochrome digital (1360 × 1024 pixel) camera mounted on a Leica Dialux compound microscope at a magnification between 40x and 1000x. Semi-thin sections were taken progressively through the eye in order to locate a standardized transverse section of the retina that incorporated the temporo-ventral fovea^31^, and both central and dorsal retinal regions. The location of the optic nerve was used as a way of standardizing the location of section to allow for comparative analysis of both treated and control eyes.

**Figure 7:**
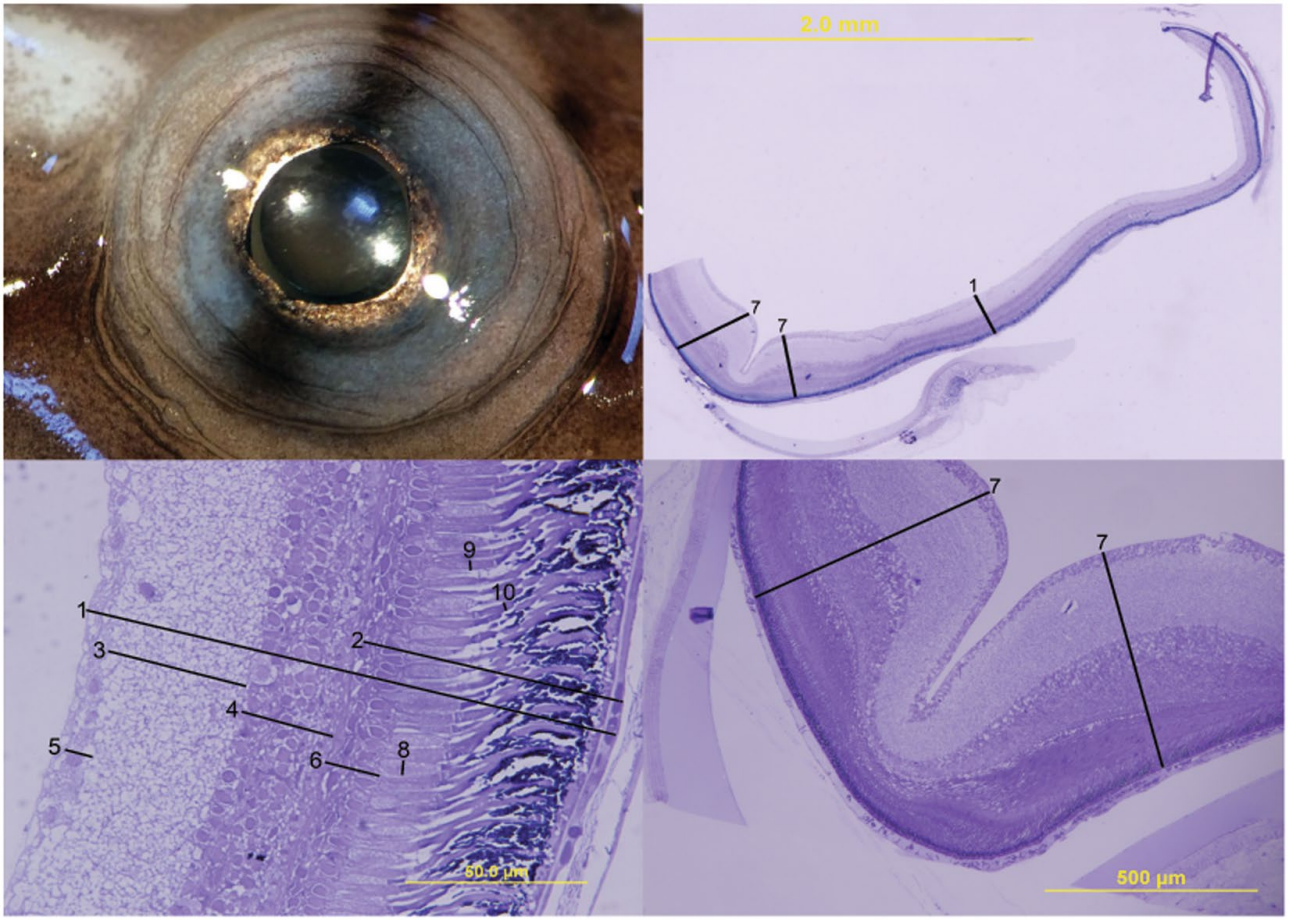
Retinal morphology characteristics measured on the eyes of *Hippocampus subelongatus*. (from top left, clockwise: Eye *in situ* prior to enucleation; Retina at x4 magnification; Fovea at x10 magnification; Retina at x100 magnification). Variables: (1) Retinal thickness, (2) Photoreceptor length, (3) Inner plexiform thickness, (4) Inner nuclear layer thickness, (5) Retinal ganglion cell layer thickness, (6) Outer nuclear layer thickness, (7) Perifoveal retinal thickness, (8) Cone photoreceptor inner segment width, (9) Rod inner segment width, (10) Rod outer segment width.

Only retinal sections that were deemed to be cut in transverse section (not obliquely) were used for analyses. Defined retinal regions (central and foveal) were examined, where retinal thickness (inner limiting membrane to Bruch’s membrane), photoreceptor length (vitread limit of synaptic terminals to sclerad limit of outer segment), rod inner and outer segment length and width, cone inner segment width, the thickness of the inner and outer nuclear layers, and the thickness of the inner plexiform and ganglion cell layers were analysed (Fig. 7). Measurements of eye anatomy and retinal layer structure (Table 4) were undertaken to identify gross anatomical changes or major histological damage attributable to light exposure^58^. The thickness of the perifoveal retinal region was also measured to provide a way of assessing any gross changes specific to the visual axis given this retinal specialization would typically be aligned and fixated on prey during feeding^31,59,60^. The perifoveal region was targeted due to the difficulty in accurately identifying the central region of the foveal pit and the elongated orientation of the foveal slit in this species.

**Table 4:** Variables measured to test effects of flash photography on the eye anatomy of *Hippocampus subelongatus*.

Variable	Measurements
Anteroposterior eye diameter (Left)	One measurement per eye
Anteroposterior eye diameter (Right)	One measurement per eye
Dorsoventral eye Diameter (Left)	One measurement per eye
Dorsoventral Diameter (Right)	One measurement per eye
Lens Diameter (Left)	One measurement per lens
Lens Diameter (Right)	One measurement per lens
Retinal thickness	Three measurements per retina
Photoreceptor length	Three measurements per retina
Inner plexiform thickness	Three measurements per retina
Inner nuclear layer thickness	Three measurements per retina
Retinal ganglion cell layer thickness	Three measurements per retina
Outer nuclear layer thickness	Three measurements per retina
Perifoveal retinal thickness	Two measurements left and right of fovea
Cone photoreceptor inner segment width	Five measurements per retina
Rod inner segment width	Five measurements per retina
Rod outer segment width	Five measurements per retina

#### Data analyses

We compared 13 variables: three variables of gross anatomy for both the eyes and lenses, and ten variables relevant to retinal anatomy for the right eyes. The sizes of different retinal structures, particularly the thickness of easily differentiated retinal layers, were measured using ImageJ^61^. For each retina, we took multiple different measurements for each retinal morphological variable and used the mean of those measurements for testing (Table 4 and Fig. 7). All metrics were measured blind; the researchers conducting measurements were unaware which treatment groups each sample belonged to.

Data from ocular gross morphology (eye and retina) and retinal morphology were analysed using the PERMANOVA+ package in Primer 7 to do a multivariate analysis of variance. For both ocular and retinal morphology, data were first normalised to allow for comparisons as measurements were on different scales. We tested correlated variables and removed variables that were strongly correlated (r > 0.85) prior to testing. Data met assumptions of normality and heterogeneity, so untransformed data was used to construct a Euclidean distance resemblance matrix. We tested the differences between treatments using PERMANOVA+ based on a one factor design (Treatment (Fixed)), with an unrestricted permutation of raw data, running 9999 permutations.

To compare variables separately, all variables were tested for assumptions of normality and the two treatment groups compared with unpaired t-tests using R^49^. For one variable (photoreceptor length) assumptions did not meet normality, so a Wilcoxon rank test was used to test differences between control and flashed groups. Holm corrections were applied to account for the increased likelihood of false positive Type I errors^48^. Original p-values are presented in results for clarity, as non-significant Holm-adjusted p-values commonly resulted in p-value equal to one.

## Supplementary information

Supporting info video

Supplementary info

## Data Availability

The datasets generated during the current study are available from the corresponding author on reasonable request.
