# Supplementary material for: Behavioural and pathomorphological impacts of flash photography on benthic fishes
Source: Sci Rep. 2019 Jan 24;9:748. doi: 10.1038/s41598-018-37356-2 (PMC6345839; doi:10.1038/s41598-018-37356-2)

Behavioural and pathomorphological impacts of flash photography on benthic fishes

Author Affiliations

Maarten De Brauwer [a*], Luke M. Gordon [a], Tanika C. Shalders [a], Benjamin J. Saunders [a], Michael Archer [b], Euan S. Harvey [a], Shaun P. Collin [b], Julian C. Partridge [c], Jennifer L. McIlwain [a]

[a] School of Molecular and Life Sciences, Curtin University, Perth, Australia
[b] Oceans Graduate School and the Oceans Institute, The University of Western Australia, Crawley, 6009, WA, Australia
[c] School of Biological Sciences and the Oceans Institute, The University of Western Australia, Crawley, 6009, WA, Australia

*Corresponding author: maarten.debrauwer@curtin.edu.au

Supplementary materials - Table 1. Results of paired Wilcoxon rank sum tests comparing controls to treatments, showing original p-values. T: combined mean of all treatments, C: control, TP: diver presence, T1: flash, T2: manipulation, T3: manipulation + flash. Significance level after Holm-Bonferroni corrections *p<0.05, **p<0.01, ***p<0.001, °p<0.1

Reaction	C – TP	C – T1	C – T2	C – T3
Antennariidae				
Movement	1	0.346	0.014*	0.022*
Turn	NA	0.345	0.006**	0.016*
Erect	NA	0.371	0.008**	0.009**
Yawn	0.414	0.345	0.371	NA
Lure	0.361	0.174	1	1
Syngnathoidei				
Turn	0.129	0.0136*	0.014*	0.014*
Feed	0.149	0.832	0.371	0.789
Hippocampus spp.				
Movement	NA	1	0.098	0.269
Solenostomus spp.				
Movement	0.250	0.250	0.098	0.174

Supplementary materials – Table 2. Results of t-test comparing different morphological variables between Hippocampus subelongatus exposed to flash photography (N = 10) and controls (N = 10).

Variable	Mean Control	SD	N	SE	Mean Flashed	SD	N	SE	t value	df	p-value
Eye size	165.10	16.723	10	5.288	168.80	18.778	10	5.938	-0.437	17.763	0.647
Eye weight	6.36	1.685	10	0.533	6.17	1.723	10	0.545	0.248	17.991	0.807
AP diameter Left	3.90	0.372	10	0.118	4.06	0.188	10	0.059	-1.259	13.324	0.230
AP Diameter Right	4.19	0.214	10	0.068	4.12	0.289	10	0.091	0.657	14.644	0.521
DV Diameter Left	4.06	0.236	10	0.075	4.04	0.216	10	0.068	0.140	17.861	0.892
DV Diameter Right	4.22	0.293	10	0.093	4.06	0.178	10	0.056	1.440	12.924	0.174
Lens Diameter Left	1.40	0.120	10	0.038	1.34	0.162	10	0.051	0.863	16.584	0.400
Lens Diameter Right	1.27	0.094	10	0.030	1.26	0.047	10	0.015	0.240	13.228	0.814
Retinal thickness	200.00	20.099	10	6.356	210.00	34.104	10	10.785	-0.783	14.579	0.446
Photoreceptor length	75.01	12.132	10	3.836	73.04	5.743	10	1.816	0.463	12.841	0.651
Photoreceptor length	75.01	12.123	10	3.834	73.04	5.743	10	1.816	49	-	0.971
Inner plexiform thickness	49.36	11.415	10	3.610	55.25	15.729	10	4.974	-0.959	16.422	0.352
Inner nuclear layer thickness	32.06	10.307	10	3.259	34.94	10.666	10	3.373	-0.614	17.979	0.547
Retinal ganglion cell layer thickness	5.93	1.730	10	0.547	6.25	1.380	10	0.436	-0.467	17.152	0.646
Outer nuclear layer thickness	12.03	3.940	10	1.246	12.35	4.351	10	1.376	-0.170	17.826	0.867
Perifoveal retinal thickness	438.80	74.949	6	30.598	399.30	24.262	6	9.905	1.227	6.037	0.266
Cone photoreceptor inner segment width	3.14	0.512	10	0.162	2.83	0.491	10	0.155	1.388	17.966	0.182
Rod inner segment width	2.23	0.419	10	0.132	2.19	0.442	10	0.140	0.218	17.949	0.830
Rod outer segment width	2.20	0.315	10	0.100	2.10	0.483	10	0.153	0.567	15.487	0.578

Figure. Treatment aquarium set-up

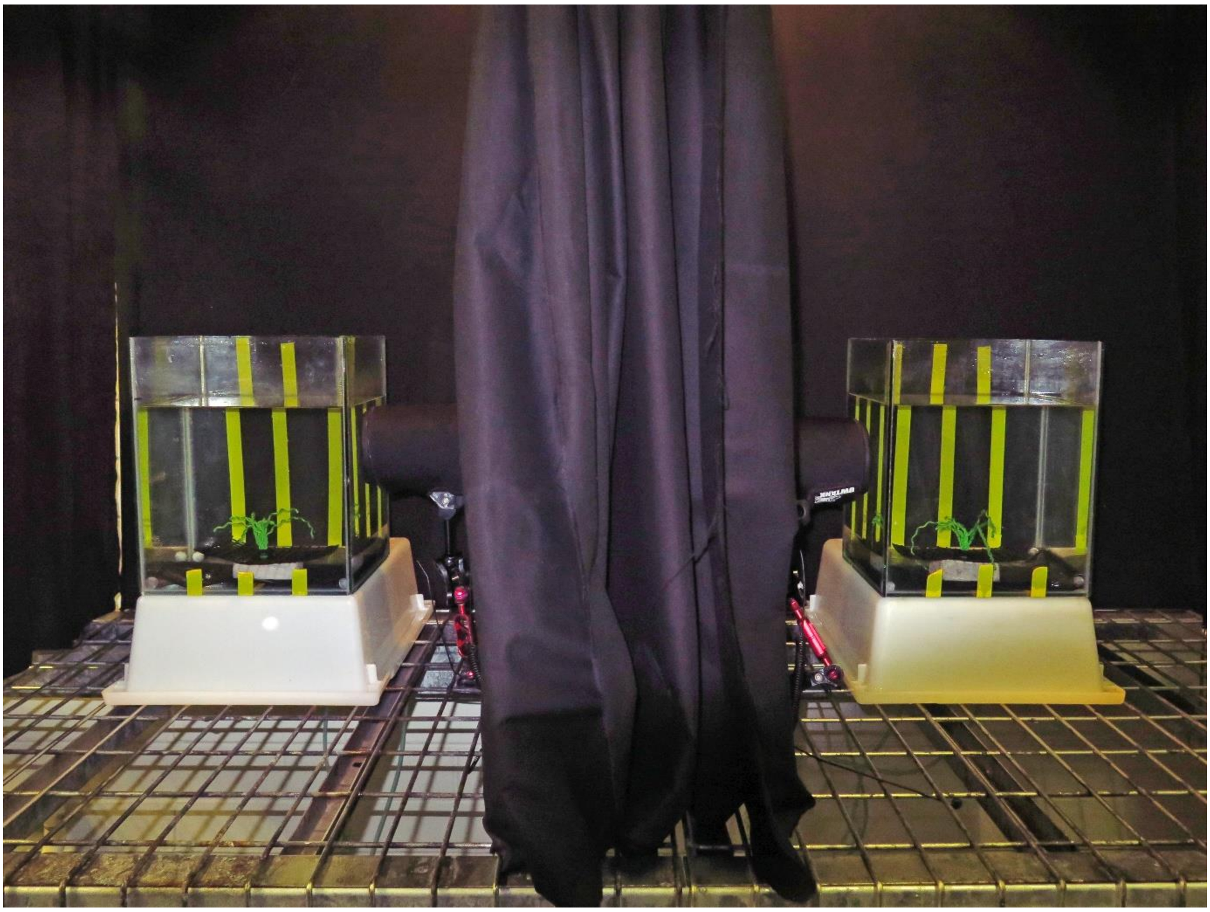

Supplement: Supplementary file 2 — Supplementary info [file 41598_2018_37356_MOESM2_ESM.pdf]
